# TRIM38 Inhibits Zika Virus by Upregulating RIG-I/MDA5 Pathway and Promoting Ubiquitin-Mediated Degradation of Viral NS3 Protein

**DOI:** 10.3390/v17020199

**Published:** 2025-01-30

**Authors:** Jing He, Yulian Kuang, Kui Xu, Rong Huang, Xiaoyao Yang, Liyao Deng, Xiaojuan Feng, Yang Ren, Jian Yang, Lei Yuan

**Affiliations:** Institute of Basic Medicine, North Sichuan Medical College, Nanchong 637100, China

**Keywords:** Zika virus, TRIM38, NS3 protein, antiviral activity, ubiquitination

## Abstract

Members of the tripartite motif (TRIM)-containing protein family play crucial roles in regulating immune system responses. The TRIM38 protein regulates host innate immunity and directly degrades some viral proteins through its E3 ubiquitin ligase activity. This study demonstrated that Zika virus (ZIKV) infection can promote the expression of TRIM38 in human glioma cells (U251). TRIM38 overexpression restricted ZIKV replication in U251 cells, while TRIM38 knockout enhanced ZIKV replication. TRIM38 overexpression upregulated the RIG-I/MDA5 pathway and promoted the level of IFN-β early during viral infection, while TRIM38 knockout had the opposite effect. In addition, TRIM38 interacts with ZIKV non-structural protein 3 (NS3) and degrades the NS3 protein through a lysosome-dependent manner via the E3 ligase activity of TRIM38. Deletion of the RING domain of TRIM38 abrogates its interaction with NS3 and impairs the antiviral activity of TRIM38. Our results indicate that TRIM38 is a novel antiviral protein against ZIKV, and it exerts antiviral activity by upregulating the RIG-I/MDA5 pathway, increasing IFN-β levels, and degrading the viral NS3 protein.

## 1. Introduction

Zika virus (ZIKV) is an important arbovirus belonging to *Orthoflavivirus* in the *Flaviviridae* family. Since its initial isolation from sentinel rhesus monkeys in Uganda in 1947, it remained a minor concern until 2016, when a significant outbreak in the Americas drew global attention. ZIKV is primarily transmitted by *Aedes aegypti* mosquitoes, and it can also be transmitted through mother-to-child transmission, blood transfusion, sexual contact, and potentially urine [1]. As of February 2023, Zika virus transmission has been recorded in 89 countries and territories globally, including the Americas, Africa, Southeast Asia, and the Western Pacific [2]. The spread of ZIKV caused panic in Latin American and Caribbean countries, with an estimated 440,000 to 1.3 million cases recorded in Brazil during the 2016 outbreak. ZIKV infection generally causes mild symptoms. However, ZIKV infection during pregnancy can lead to miscarriage and severe congenital anomalies, such as microcephaly in newborn infants. Additionally, ZIKV infection has been associated with the development of Guillain–Barré syndrome in adults [3]. Due to the antibody-dependent enhancement (ADE) reaction, there are currently no approved vaccines for ZIKV, and few vaccine candidates are undergoing clinical trials [4].

ZIKV has a single-stranded RNA genome containing a 5′ untranslated region (UTR) (UTR), followed by a 10,272-nucleotide coding region and a 3′ UTR. The only open reading frame (ORF) codes a large polyprotein that can be cleaved into three structural proteins (C, prM, and E) and seven non-structural proteins (NS1, NS2A, NS2B, NS3, NS4A, NS4B, and NS5) by viral and cellular proteases. ZIKV is divided into two major genotypes (African and Asian), with the majority of outbreaks being attributed to the Asian genotype [5].

Currently, more than 80 distinct TRIM proteins have been identified, which regulate a variety of cellular processes, including apoptosis, antiviral immunity, inflammatory response, autophagy, and tumorigenesis [6]. The antiviral effects of TRIM proteins include interaction with viral proteins and regulation of host innate immunity. TRIM proteins affect antiviral innate immunity mainly by regulating the RIG-I-like receptors (RLRs), DNA sensor cGAS, Toll-like receptors (TLRs), and IFNα/β receptor (IFNAR) signaling pathways [7]. Members of the TRIM family have a conserved domain architecture, from the N to the C terminus, consisting of a RING, one or two B-boxes (B1 box, B2 box), and a coiled-coil domain [8]. The RING domain of most TRIM proteins has E3 ubiquitin ligase activity, and some TRIM proteins can degrade viral proteins via the ubiquitin–proteasome pathway [9]. TRIM38 contains a RING, two B-boxes, a coiled-coil, and a C-terminal PRY-SPRY domain. TRIM38 expression can be induced by Toll-like receptor (TLR) ligands, type I interferons (IFN-I), and viral infections, suggesting that TRIM38 may function as an interferon-stimulated gene (ISG). TRIM38 positively regulates the induction of downstream antiviral genes mediated by MDA5 and RIG-I. As an E3 SUMO1 ligase for MDA5 and RIG-I, TRIM38 catalyzes the sumoylation of MDA5 at K43/K865 and RIG-I at K96/K889. The sumoylation of MDA5 at K43 and RIG-I at K96 is essential for the effective initiation of the innate immune response to RNA viruses. TRIM38 is also a SUMO ligase for cGAMP synthase (cGAS) and a stimulator of interferon genes (STING). The sumoylation of cGAS and STING dynamically regulates the innate immune response to DNA virus infections [10]. TRIM38 knockout mice infected with vesicular stomatitis virus (VSV), encephalomyocarditis virus (EMCV), or herpes simplex virus type 1 (HSV-1) had significantly higher viral titers in the brain and a higher mortality rate compared to wild-type mice, indicating that TRIM38 plays a crucial role in host defense against both RNA and DNA viruses [11,12]. Studies have shown that TRIM38 overexpression inhibits hepatitis B virus (HBV) replication and viral gene expression by enhancing the expression of antiviral proteins [13].

At present, the effect of TRIM38 on ZIKV replication has not been reported. In this study, we identified that TRIM38 could restrict ZIKV replication in vitro. TRIM38 could upregulate the RIG-I/MDA5 pathway and promote the level of IFN-β early during viral infection. We found that the antiviral function of TRIM38 against ZIKV is dependent on its ubiquitin E3 ligase activity. Furthermore, our findings suggest that TRIM38 interacts with the ZIKV NS3 protein and facilitates its degradation via a lysosome-dependent pathway.

## 2. Materials and Methods

### 2.1. Cell Lines, Viruses and Antibodies

Human embryo kidney (HEK-293T), human glioma (U251), and African green monkey kidney (Vero) cells were purchased from the American Type Culture Collection (ATCC) and cultured in Dulbecco’s modified Eagle medium (DMEM) supplemented with 10% fetal bovine serum (FBS) at 37 °C with 5% CO_2_. The ZIKV PRVABC59 strain (GenBank NO. KX087101) was generated using reverse genetics technology in the Arbovirus Laboratory of North Sichuan Medical College (Nanchong City, China). Anti-ZIKV E protein rabbit polyclonal antibody (pAb) and anti-ZIKV C protein rabbit pAb were purchased from GeneTex (Irvine, CA, USA). Anti-NAK/TBK1 (phospho S172) rabbit monoclonal antibody (mAb) was purchased from Abcam (Waltham, MA, USA). Anti-TRIM38 rabbit pAb, anti-MDA5 rabbit pAb, anti-ISG56 rabbit pAb, anti-OAS1 rabbit pAb, anti-OAS3 rabbit pAb, anti-ubiquitin rabbit pAb, anti-Myc tag mouse mAb, anti-Myc tag rabbit pAb, anti-Flag tag mouse mAb, and anti-Flag rabbit pAb were purchased from ProteinTech (Wuhan, China). Anti-phospho-IRF3-s396 rabbit mAb was purchased from ABclonal (Wuhan, China). Anti-OAS2 rabbit pAb and anti-ISG15 rabbit pAb were purchased from Immunoway (SuZhou, China). Anti-RIG-I rabbit pAb was purchased from BBI (Shanghai, China), and anti-GAPDH rabbit mAb was purchased from Beyotime (Shanghai, China).

### 2.2. Detection of TRIM38 Expression

U251 cells at about 70% confluence in 6-well plates were infected with the ZIKV PRVABC59 strain (multiplicity of infection, MOI = 1) or treated with different concentrations of IFN-β. The cells were harvested at 12, 24, 36, and 48 h post-infection (hpi) or 36 h after treatment with IFN-β, and total RNA was extracted for quantitative RT-PCR using primers targeting the coding regions of TRIM38. In addition, the lysates of U251 cells were collected to detect the TRIM38 protein by Western blotting. Uninfected and untreated cells served as the mock controls.

### 2.3. TRIM38 Overexpression and Knockout

The full-length coding region sequence of human TRIM38 amplified by PCR was inserted into pLVx-Flag-puro plasmid to construct the pLVx-Flag-TRIM38-puro plasmid. The sgRNA sequences targeting TRIM38 were designed using online software (http://chopchop.cbu.uib.no, accessed on 12 July 2023). The double-stranded sgRNAs (sgTRIM38 F: 5′-CACCGTGTCGGGCTCCATTTCATA-3′, R: 5′-AAACTATGAAATGGAGCCCGACAC-3′) were synthesized. The sgRNA sequence was inserted into the *BsmBI* site of the plasmid p-lentiCRISPR-V2 to form the plasmid p-lentiCRISPR-V2-sgTRIM38. The pLVx-Flag-TRIM38-puro (8 μg) or p-lentiCRISPR-V2-sgTRIM38 (8 μg) was co-transfected with the packaging plasmids (6 μg psPax2 and 4 μg pMD2.G) into 293T cells in a 10 cm petri dish for generation of overexpression lentivirus LV-Flag-TRIM38 or knockout lentivirus LV-TRIM38-KO. U251 cells were transduced with lentivirus and cultured in DMEM medium containing polybrene (5 μg/mL) and 10% fetal bovine serum for 48 h, then puromycin (3 μg/mL) was added for selection. After three rounds of selection, the protein was extracted and the overexpression or knockout of TRIM38 was verified by Western blotting. U251 cells transduced with the empty vector lentiviruses LV-Flag or LV-V2 were used as negative controls. Cells were infected with the ZIKV PRVABC59 strain and then cultured in DMEM medium containing 2% fetal bovine serum. Samples were collected at different time points after culture and detected by quantitative RT-PCR, plaque assay, Western blotting, and immunofluorescence assay (IFA).

### 2.4. Quantitative RT-PCR

After infection with ZIKV, the cells and supernatants were harvested and freeze-thawed once, then total RNA was extracted for quantitative RT-PCR targeting the E gene of ZIKV. TRIM38 overexpressing and knockout cells were infected with ZIKV (MOI = 1). Cells were harvested at different time points after infection, and total RNA was extracted for quantitative RT-PCR targeting the IFN-β, RIG-I, MDA5, TBK1, IRF3, ISG15, ISG56, OAS1, OAS2, and OAS3 genes. GAPDH served as the housekeeping gene. Primers are listed in Table 1.

### 2.5. Growth Curve of ZIKV

U251 cells were infected with ZIKV (MOI = 1). After 1 h of adsorption at 37 °C, the supernatants were removed. Subsequently, 10 mL of DMEM containing 2% FBS was added, and 150 µL of supernatant was collected every 12 h post-infection. The viral titers of each sample were determined by plaque assay on Vero cells.

### 2.6. Plaque Assay

The supernatants and cells were frozen and thawed once to lyse the cells and release the virus, and then centrifuged at 8000× *g* for 10 min to collect the supernatants. Vero cells at about 80% confluence in 6-well plates were incubated with serial 10-fold diluted samples. The supernatants were removed after 1 h of absorption, and 2 mL DMEM containing 2% FBS and 1% methylcellulose was added to each well. After 5 days of incubation, the supernatants were removed, and cells were stained with crystal violet dye containing 2% formaldehyde for 15 min. The number of plaques was counted to determine the titers of ZIKV.

### 2.7. Western Blotting

The cells were lysed with RIPA buffer on ice for 30 min, then the supernatant was collected after centrifugation (4 °C, 12,000× *g*, 15 min). Protein was quantified using the Bradford assay. Equal quantities of protein were separated by SDS-PAGE and then transferred onto a PVDF membrane. The membrane was blocked with 5% (*w*/*v*) bovine serum albumin (BSA) for 1 h and incubated with the primary antibody at 4 °C overnight. Then, after being washed 4 times with TBST, the membrane was incubated with a secondary antibody for 1 h and then washed with TBST 4 times. Subsequently, the western ECL substrate (Bio-Rad, Hercules, CA, USA) was added, and the protein signals were detected using a Bio-Rad ChemiDoc XRS system. GAPDH served as the internal control.

### 2.8. Co-IP Assay

The plasmids were co-transfected into 293T cells in a 10 cm dish using Lipofectamine 3000 (Thermo Fisher Scientific, Waltham, MA, USA). After 36 h of incubation, the cells were lysed with NP-40 Lysis Buffer (Beyotime, Shanghai, China) containing 50 mM Tris (pH 7.4), 150 mM NaCl, 1% NP-40, and PMSF, and the supernatants were collected after centrifugation. Anti-Myc or anti-Flag antibody (1 μg) was mixed with 40 μL magnetic beads (Beyotime, Shanghai, China) and incubated at 4 °C for 8 h. The beads were then washed 3 times with TBST. The total cell lysate was incubated with the anti-Myc or anti-Flag antibody-bound magnetic beads overnight at 4 °C with gentle shaking, and then the beads were washed 4 times with TBST. The bound proteins were eluted with SDS-PAGE loading buffer by boiling and then subjected to Western blotting.

### 2.9. Confocal Immunofluorescence Microscopy

The 293T cells on coverslips (diameter 18 mm) in 12-well plates were transfected with the 0.5 μg pLVx-Flag-TRIM38-puro and 0.5 μg pCMV-Myc-NS3 plasmid. After 24 h of incubation, the cells were washed with PBS, fixed with 4% paraformaldehyde for 30 min, and permeabilized with 0.5% Triton X-100 for 10 min. The cells were blocked with 1% BSA at room temperature for 1 h, washed with PBS, incubated with anti-Myc antibody and anti-Flag antibody at 37 °C for 2 h, and then washed 3 times with PBS. The cells were incubated with Alexa Fluor 488-labeled goat anti-mouse antibodies and Cy3-conjugated goat anti-rabbit antibodies for 2 h. After three washes with PBS, the cells were stained with DAPI for 5 min. Fluorescent images were acquired using a laser confocal microscope (Olympus FV3000, Tokyo, Japan).

### 2.10. Statistical Analyses

The quantitative RT-PCR and plaque assay were repeated three times, and the Western blotting, Co-IP assay, and immunofluorescence assay were repeated at least twice. Data were presented as the mean ± standard deviation (SD). Statistical analyses were performed using one-way ANOVA, two-way ANOVA, or Student’s *t*-test. Asterisks indicate statistical significance (ns *p* > 0.05, * *p* < 0.05, ** *p* < 0.01, *** *p* < 0.001). Figures and statistical analyses were generated using GraphPad Prism 8.0 software.

## 3. Results

### 3.1. TRIM38 Expression Is Upregulated by ZIKV Infection and IFN-β Stimulation

**Figure 1:**
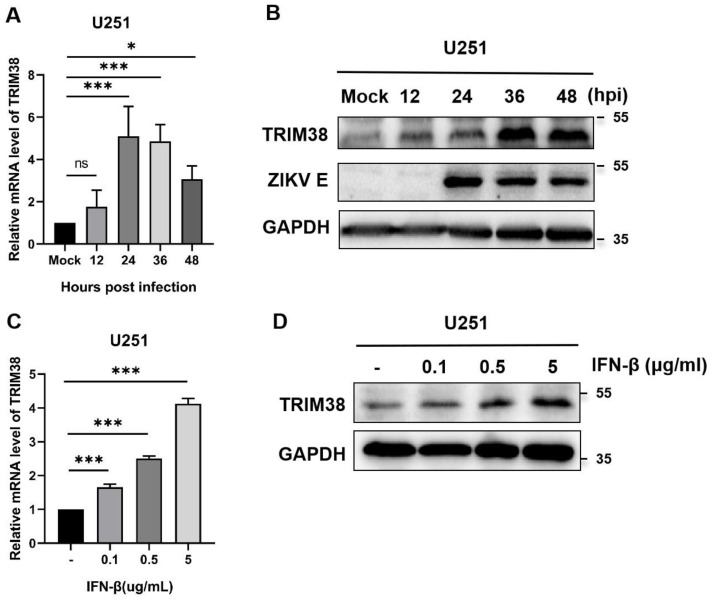
ZIKV infection and IFN-β stimulation upregulate the levels of TRIM38. (**A**) TRIM38 mRNA levels in U251 cells were quantified by qRT-PCR at 12, 24, 36, and 48 hpi with ZIKV (MOI = 1), and uninfected cells served as the mock. (**B**) E protein of ZIKV and TRIM38 protein were detected by Western blotting. (**C**,**D**) The levels of TRIM38 mRNA and protein in U251 cells were stimulated by increasing doses of IFN-β. GAPDH served as an internal control. Data are expressed as mean ± SD from three independent experiments (n = 3, ns *p* > 0.05, * *p* < 0.05, *** *p* < 0.001).

The results of quantitative RT-PCR showed that ZIKV infection significantly upregulated the transcription of TRIM38 in U251 cells. Compared with uninfected cells, the levels of TRIM38 mRNA in infected cells were increased by 5.1- (24 hpi), 4.9- (36 hpi), and 3.1-fold (48 hpi), respectively (Figure 1A). Meanwhile, the Western blotting revealed the upregulation of TRIM38 protein expression in infected cells (Figure 1B). In addition, the levels of TRIM38 mRNA and protein were significantly upregulated in a dose-dependent manner 36 h after IFN-β stimulation (Figure 1C,D).

### 3.2. TRIM38 Overexpression Inhibits ZIKV Replication

**Figure 2:**
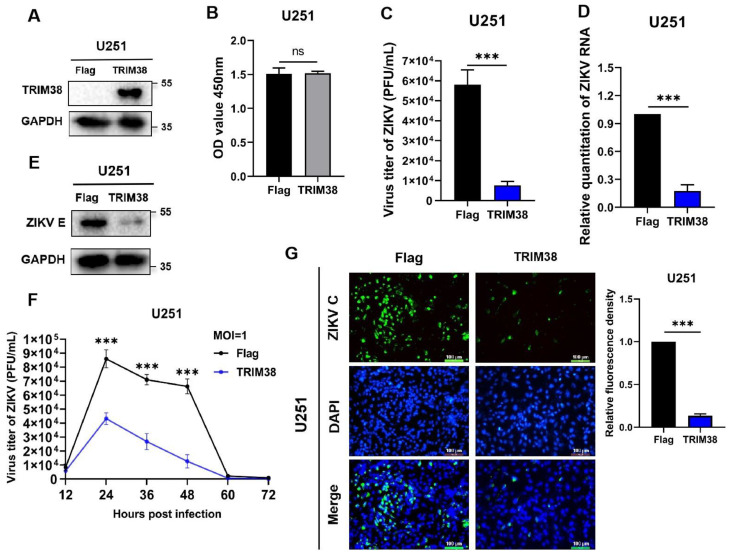
TRIM38 overexpression inhibits ZIKV replication. U251 cells with or without TRIM38 overexpression were infected with ZIKV (MOI = 1). Viral replication was measured by plaque assay, qRT-PCR, and Western blotting at 48 hpi. (**A**) TRIM38 overexpression was determined by Western blotting. (**B**) The viability of U251 cells with or without TRIM38 overexpression was analyzed using CCK8 assay. (**C**) Viral titers in U251 cells were measured by plaque assay. (**D**) Viral RNA loads in U251 cells were quantified by qRT-PCR. (**E**) Cell lysates were collected, and the ZIKV E protein was detected by Western blotting. GAPDH served as an internal control. (**F**) Growth curves of ZIKV in U251 cells were measured from 12 to 72 h post-infection by plaque assay. (**G**) U251 cells were infected with ZIKV for 48 h, and subsequently incubated with rabbit anti-ZIKV C protein antibodies, followed by incubation with Alexa Fluor 488-labeled goat anti-rabbit antibodies. Nuclei were stained with DAPI. Scale bar = 100 μm. Images were obtained using a fluorescence microscope (LEICA DMI4000 B, Wetzlar, Germany). The relative fluorescence density of green fluorescence was quantified using DAPI-stained cells as an internal reference. Data are expressed as mean ± SD from three independent experiments (n = 3, ns *p* > 0.05, *** *p* < 0.001).

To evaluate the antiviral effect of TRIM38 against ZIKV, U251 cells overexpressing TRIM38 were established by transduction with lentivirus LV-Flag-TRIM38 (Figure 2A). Cells transduced with lentivirus LV-Flag served as the negative control. The results showed that there was no significant difference in the viability between the overexpressing cells and the negative control cells (Figure 2B). U251 cells with or without TRIM38 overexpression were infected with ZIKV (MOI = 1). Then, the viral replication was measured at 48 hpi using plaque assay, qRT-PCR, and Western blotting. The results showed that TRIM38 overexpression significantly reduced both viral titers and viral RNA loads. Specifically, viral titers were reduced by 7.6-fold (Figure 2C), while viral RNA loads were reduced by 85.6% (Figure 2D). Western blotting results showed that viral replication was significantly inhibited by TRIM38 overexpression (Figure 2E). The growth curve of ZIKV indicated that TRIM38 overexpression significantly suppressed ZIKV replication from 24 to 48 h post-infection, with the most pronounced inhibitory effect observed at 48 hpi (Figure 2F). The immunofluorescence analysis further demonstrated that TRIM38 effectively suppresses ZIKV replication (Figure 2G).

### 3.3. TRIM38 Knockout Enhances ZIKV Replication

**Figure 3:**
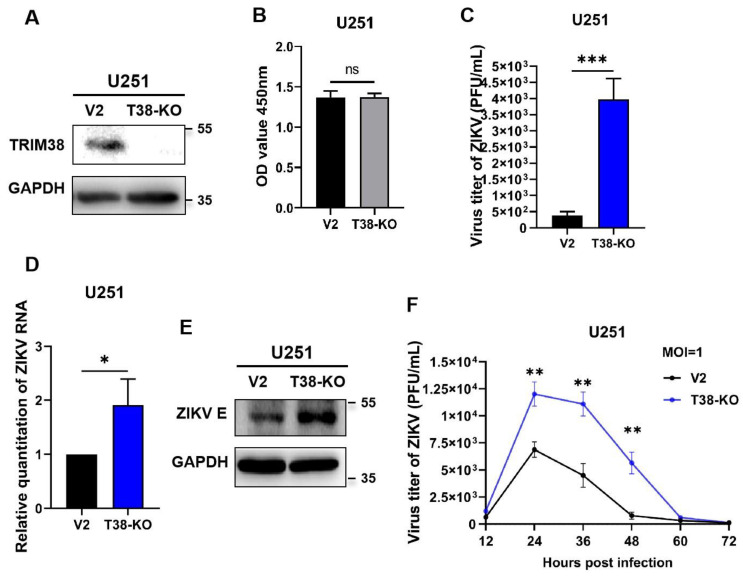
TRIM38 knockout enhances ZIKV replication. TRIM38 knockout U251 cells and negative control cells (V2) were infected with ZIKV (MOI = 1). Viral replication was measured by plaque assay, qRT-PCR, and Western blotting at 48 hpi. (**A**) Blocking of TRIM38 expression was determined by Western blotting. (**B**) The viability of U251 knockout cells and negative control were analyzed using CCK8 assay. (**C**) Viral titers in U251 cells were measured by plaque assay. (**D**) Viral RNA loads in U251 cells were quantified by qRT-PCR. (**E**) Cell lysates were collected at 48 hpi, and the ZIKV E protein was detected by Western blotting. GAPDH served as an internal control. (**F**) Growth curves of ZIKV in U251 cells were measured from 12 to 72 h post-infection by plaque assay. Data are expressed as mean ± SD from three independent experiments (n = 3, ns *p* > 0.05, * *p* < 0.05, ** *p* < 0.01, *** *p* < 0.001).

To further determine the influence of TRIM38 on ZIKV replication, TRIM38 knockout U251 cells were established by transduction with lentivirus LV-TRIM38-KO (Figure 3A). Cells transduced with lentivirus LV-V2 served as the negative control. The results of the CCK8 assay showed that there was no significant difference in viability between the knockout cells and the negative control (Figure 3B). Knockout cells and negative control were infected with ZIKV (MOI = 1). Then, the viral replication was measured at 48 hpi by plaque assay, qRT-PCR, and Western blotting. The results showed that the viral titers and viral RNA loads in TRIM38 knockout cells increased by 10.4-fold and 1.9-fold, respectively (Figure 3C,D). The Western blotting results demonstrated enhanced replication of ZIKV in the knockout cells (Figure 3E). The growth curve indicated that TRIM38 knockout significantly enhanced ZIKV replication from 24 to 48 hpi, with the most pronounced effect observed at 48 hpi (Figure 3F).

### 3.4. TRIM38 Upregulates the RIG-I/MDA5 Pathway

**Figure 4:**
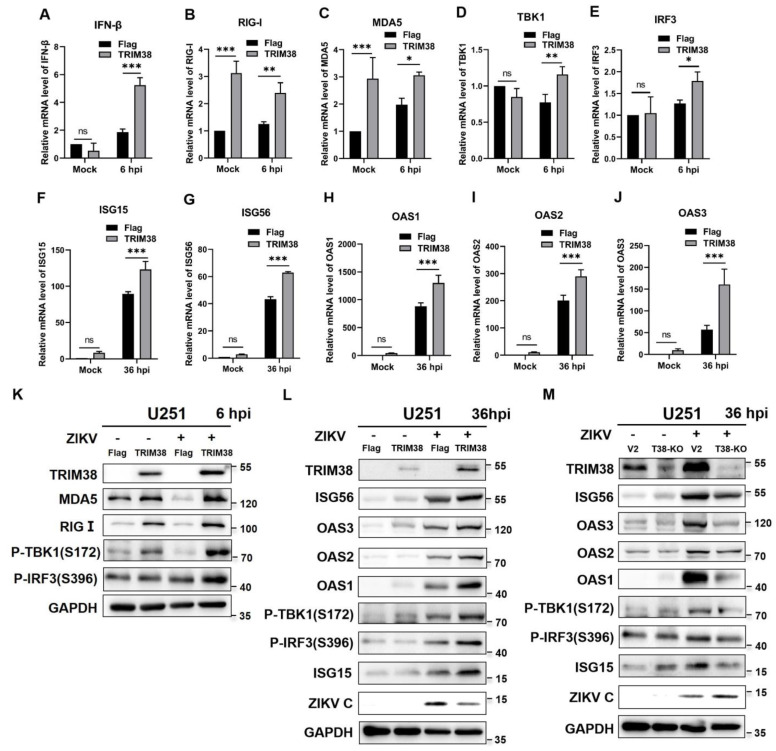
TRIM38 positively regulates the RIG-I/MDA5 pathway. U251 cells with or without TRIM38 overexpression were infected with ZIKV (MOI = 1). Then, the mRNA and protein levels were measured by qRT-PCR and Western blotting. Uninfected cells served as the mock controls. (**A**–**E**) Total RNA of cell cultures was extracted at 6 hpi, and the mRNA levels of IFN-β, RIG-I, MDA5, TBK1, and IRF3 were quantified by qRT-PCR. (**K**) Cell lysates were collected at 6 hpi, and the proteins were detected by Western blotting. (**F**–**J**,**L**) At 36 hpi, the mRNA and protein levels of ISG15, ISG56, OAS1, OAS2, and OAS3 in U251 cells were measured by qRT-PCR and Western blotting. (**M**) The levels of these proteins in TRIM38 knockout cells were measured using Western blotting. GAPDH served as an internal control. Data are expressed as mean ± SD from three independent experiments (n = 3, ns *p* > 0.05, * *p* < 0.05, ** *p* < 0.01, *** *p* < 0.001).

To determine the regulatory effect of TRIM38 on the RIG-I/MDA5 pathway, U251 cells with or without TRIM38 overexpression in 6-well plates were infected with ZIKV (MOI = 1). At 6 hpi and 36 hpi, the total RNA of cell cultures was extracted for qRT-PCR, while cell lysates were collected for Western blotting. Uninfected cells served as the mock controls. The results of qRT-PCR showed that the IFN-β, RIG-I, MDA5, TBK1, and IRF3 mRNA levels in TRIM38 overexpressing cells increased by 2.8-, 1.9-, 1.6-, 1.5-, and 1.5-fold, respectively, compared with those in the negative control cells at 6 hpi. In uninfected cells, TRIM38 overexpression led to a 3.1-fold and 2.9-fold increase in RIG-I and MDA5 mRNA levels, respectively (Figure 4A–E). Western blotting results showed that TRIM38 overexpression significantly enhanced the expression levels of RIG-I and MDA5 in both infected and uninfected cells. The expression levels of TBK1 and IRF3 were significantly increased in TRIM38 overexpressing cells after infection with ZIKV (Figure 4K). At 36 hpi, the mRNA levels of ISG15, ISG56, OAS1, OAS2, and OAS3 in TRIM38 overexpressing cells increased by approximately 1.4-, 1.5-, 1.5-, 1.5-, and 2.8-fold, respectively. These genes were expressed at low levels in uninfected cells (Figure 4F–J). Western blotting results revealed that in infected cells, TRIM38 overexpression increased the expression of these proteins, while TRIM38 knockout decreased their expression. The expression of these proteins did not change significantly in uninfected cells (Figure 4L,M). These results indicated that TRIM38 positively regulates the RIG-I/MDA5 pathway and promotes the production of IFN-β early during viral infection.

### 3.5. TRIM38 Interacts with the ZIKV NS3 Protein, Mediating Its Degradation Through a Lysosome-Dependent Pathway

**Figure 5:**
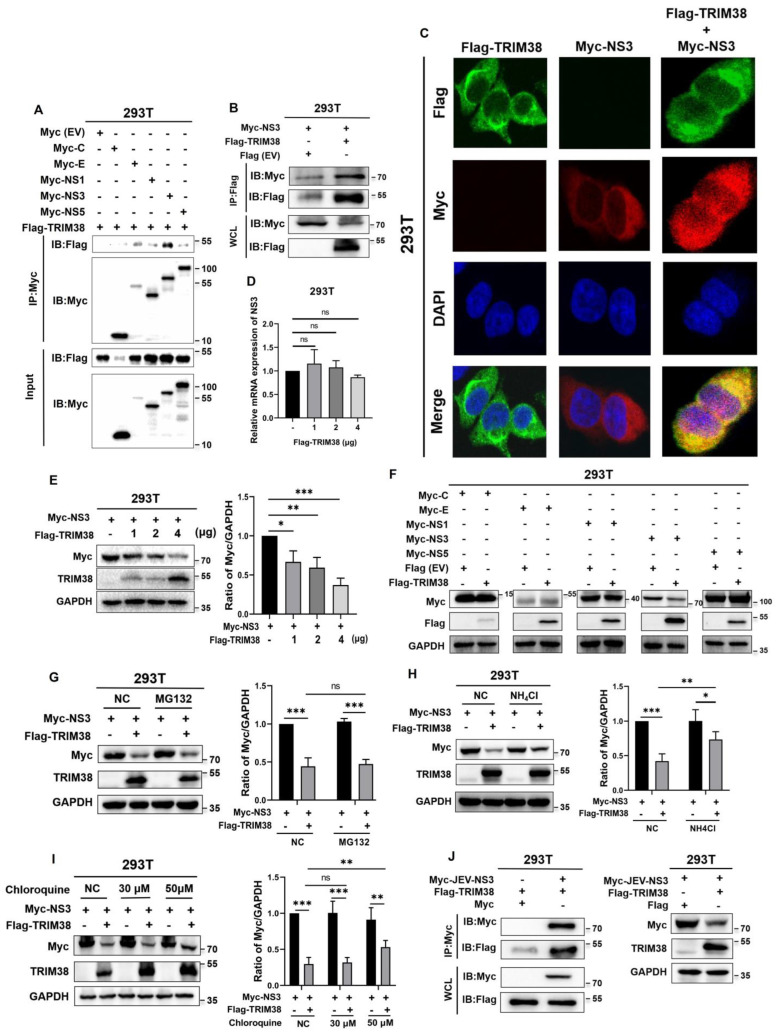
TRIM38 interacts with NS3 protein and mediates NS3 degradation via a lysosome-dependent pathway. 293T cells in a 10 cm dish were co-transfected with plasmid Flag-TRIM38 and plasmids expressing Myc-tagged ZIKV viral proteins. The cell lysates were collected at 36 hpt for Co-IP assay. (**A**) Immunoprecipitation (IP) was performed using the anti-Myc rabbit polyclonal antibody, followed by Western blotting using the anti-Flag mouse monoclonal antibody and the anti-Myc mouse monoclonal antibody. WCL, whole cell lysates. (**B**) IP was performed using the anti-Flag mouse monoclonal antibody, followed by Western blotting using the anti-Flag rabbit polyclonal antibody and the anti-Myc rabbit polyclonal antibody. (**C**) Co-localization of TRIM38 with NS3. 293T cells were co-transfected with plasmids Flag-TRIM38 and Myc-NS3. The cells were fixed at 24 hpt and were subjected to immunofluorescence assay using anti-Flag and anti-Myc antibodies to detect Flag-TRIM38 (green) and Myc-NS3 (red). The nucleus was stained with DAPI (blue). Images were obtained using a laser confocal microscope (Olympus FV3000, Tokyo, Japan). (**D**,**E**) TRIM38 mediates NS3 degradation. 293T cells in a 6 cm dish were co-transfected with a fixed amount of Myc-NS3 (3 μg) and increasing amounts of Flag-TRIM38, equalizing the DNA doses with empty vector. The mRNA and protein levels of NS3 were evaluated by qRT-PCR and Western blotting, respectively, at 36 hpt. GAPDH served as an internal control. (**F**) TRIM38 specifically promotes NS3 degradation. 293T cells in a 6 cm dish were co-transfected with plasmids Flag-TRIM38 and Myc-C, E, NS1, NS3, or NS5. At 36 hpt, the cells were harvested for Western blotting. (**G**–**I**) 293T cells in a 6 cm dish were co-transfected with Flag-TRIM38 (3 μg) and Myc-NS3 (3 μg). MG132 (10 μM), NH_4_Cl (30 mM), chloroquine (30 μM and 50 μM), or DMSO (negative control, NC) was added at 36 hpt, and the cells were harvested after 6 h of incubation. The levels of NS3 protein were evaluated by Western blotting. GAPDH served as an internal control, and the relative quantification of the detected signal was analyzed using ImageJ software. Data are expressed as mean ± SD from three independent experiments (n = 3, ns *p* > 0.05, * *p* < 0.05, ** *p* < 0.01, *** *p* < 0.001). (**J**) 293T cells in a 10 cm dish were co-transfected with Flag-TRIM38 and Myc-JEV NS3 or empty vector. Cell lysates were collected at 36 hpt for the Co-IP assay. Data are representative of two independent experiments.

To determine the specific interaction of TRIM38 with viral proteins, 293T cells in a 10 cm dish were co-transfected with plasmid pLVx-Flag-TRIM38-puro and plasmids expressing Myc-tagged ZIKV viral proteins. After 36 h of incubation, the cell lysates were collected for Co-IP assay. The results demonstrated that TRIM38 interacts with the ZIKV NS3 protein (Figure 5A,B). Furthermore, the immunofluorescence assay results showed that TRIM38 and ZIKV NS3 localize in the same cellular compartment in 293T cells, and they co-localize in the cytoplasm (Figure 5C). To investigate whether TRIM38 could influence the stability of ZIKV NS3 protein, 293T cells in a 6 cm dish were co-transfected with a fixed amount of pCMV-Myc-NS3 (3 μg) and with increasing amounts of Flag-TRIM38 (1, 2 and 4 μg), equalizing the DNA doses with the empty vector (Flag (EV)). The protein and mRNA levels of NS3 were analyzed by Western blotting and qRT-PCR, respectively, at 36 h post-transfection (hpt). The results showed that increasing doses of TRIM38 reduced the NS3 protein level without affecting its mRNA levels (Figure 5D,E). To confirm the specificity of TRIM38 in promoting NS3 degradation, 293T cells were co-transfected with the plasmid Flag-TRIM38 and overexpression plasmids encoding ZIKV C, E, NS1, NS3, or NS5 proteins. The cells were harvested for Western blotting at 36 hpt. The results showed that TRIM38 could promote the degradation of NS3, but had no significant effect on the other viral proteins (Figure 5F). To further explore the pathway through which TRIM38 mediates NS3 degradation, 293T cells in a 6 cm dish were co-transfected with Flag-TRIM38 (3 μg) and Myc-NS3 (3 μg). At 36 hpt, proteasome inhibitor MG132 (10 μM), lysosome inhibitor NH_4_Cl (30 mM), lysosome inhibitor chloroquine (30 and 50 μM), or dimethyl sulfoxide (DMSO) was added. After a 6-h incubation, the cells were harvested. Western blotting results revealed that the levels of NS3 protein were markedly reduced upon overexpression of TRIM38 in the presence of DMSO and MG132 treatments. Conversely, the levels of NS3 protein were restored following treatment with NH4Cl (30 mM) and chloroquine (50 μM) (Figure 5G–I), indicating that TRIM38 mediates the degradation of NS3 via a lysosome-dependent pathway. In addition, we found that TRIM38 can also interact with the NS3 protein of the Japanese encephalitis virus (JEV) and promote its degradation (Figure 5J).

### 3.6. RING Domain Is Essential for the Interaction Between TRIM38 and ZIKV NS3, and for Inhibiting ZIKV Replication

**Figure 6:**
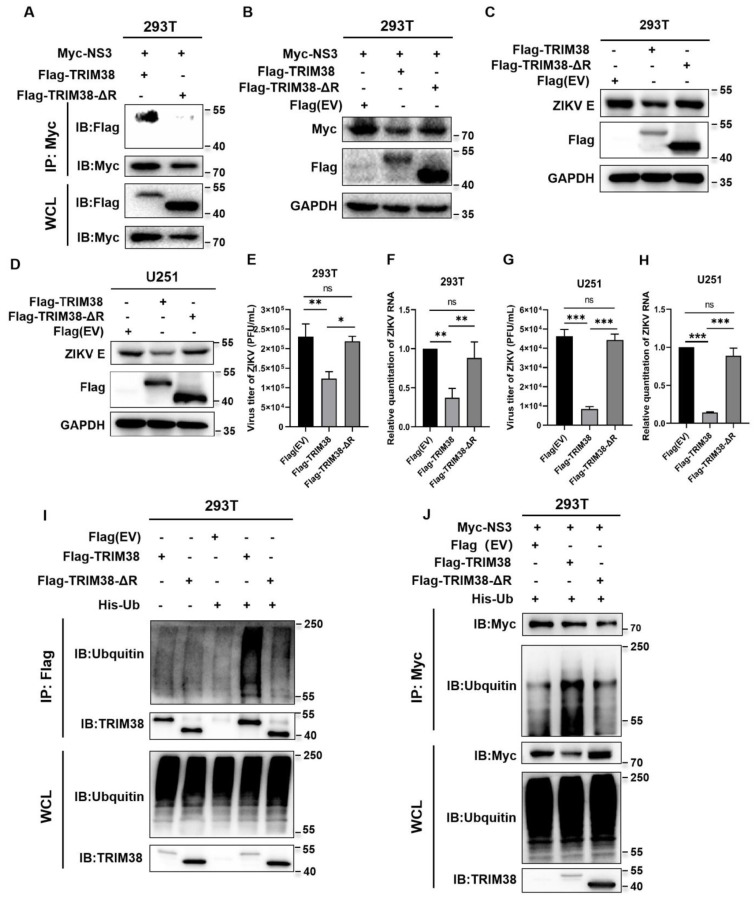
RING domain is essential for the interaction between TRIM38 and ZIKV NS3, and for inhibiting ZIKV replication. (**A**) The 293T cells in a 10 cm dish were co-transfected with Myc-NS3 and either Flag-TRIM38 or Flag-TRIM38-ΔRING. The cell lysates were collected at 36 hpt for Co-IP assay. (**B**) 293T cells in a 6 cm dish were co-transfected with plasmid Myc-NS3 (3 μg) plus the plasmids Flag-TRIM38 (3 μg), Flag-TRIM38-ΔRING (3 μg), or empty vector (3 μg). Cell lysates were collected at 36 hpt for Western blotting. (**C**,**D**) 293T and U251 cells in 6-well plates were co-transfected with plasmids Flag-TRIM38 (2 μg), Flag-TRIM38-ΔRING (2 μg), or empty vector (2 μg). The cells were infected with ZIKV (MOI = 1) at 24 hpt, and then cell lysates were collected 48 h later for Western blotting. (**E**–**H**) Viral titers and viral RNA loads in 293T and U251 cells were measured by plaque assay and qRT-PCR. GAPDH served as an internal control. Data are expressed as mean ± SD from three independent experiments (n = 3, ns *p* > 0.05, * *p* < 0.05, ** *p* < 0.01, *** *p* < 0.001). (**I**) 293T cells were co-transfected with Flag-TRIM38 or Flag-TRIM38-ΔRING, with or without His-Ub. The cell lysates were collected at 36 hpt, and ubiquitination of TRIM38 was detected by Co-IP assay. (**J**) 293T cells in a 10 cm dish were co-transfected with plasmid Myc-NS3 (5 μg) plus Flag-TRIM38 (5 μg), Flag-TRIM38-ΔRING (5 μg), or empty vector (5 μg), in the presence or absence of His-Ub (5 μg). The cell lysates were collected, and ubiquitination of NS3 was detected by Co-IP assay at 36 hpt. Data are representative of two independent experiments.

Generally, the E3 ligase activity of most TRIM proteins depends on the RING domain. Therefore, we constructed a TRIM38 mutant with RING deletion, Flag-TRIM38-ΔRING (deletion from residues 9 to 66). Co-IP results showed that TRIM38 with RING deletion failed to interact with NS3 (Figure 6A). 293T cells were co-transfected with plasmid Myc-NS3 along with the plasmids Flag-TRIM38, Flag-TRIM38-ΔRING, or empty vector. Cell lysates were collected at 36 hpt for Western blotting. The results showed that TRIM38-ΔRING led to a significantly diminished degradation of NS3 compared with wild-type TRIM38 (Figure 6B). In addition, we further determined the inhibitory effect of TRIM38-ΔRING on ZIKV replication. In 293T and U251 cells, TRIM38-ΔRING exhibited no significant antiviral activity against ZIKV compared with wild-type TRIM38 (Figure 6C–H). These results indicated that the RING domain is essential for the interaction between TRIM38 and ZIKV NS3, and for inhibiting ZIKV replication.

### 3.7. Self-Ubiquitination of TRIM38 in 293T Cells

To evaluate the E3 ubiquitin ligase activity of the RING domain of TRIM38, 293T cells were co-transfected with Flag-TRIM38 or Flag-TRIM38-ΔRING with or without His-Ub. Cell lysates were collected at 36 hpt for the Co-IP assay. The results showed that ubiquitination of TRIM38 was detected when Flag-TRIM38 was co-transfected with His-Ub. However, no significant ubiquitination was detected with TRIM38-ΔRING compared to wild-type TRIM38 (Figure 6I). These results indicated that TRIM38 self-ubiquitinates in 293T cells and that the RING domain of TRIM38 is essential for its E3 ligase activity.

### 3.8. TRIM38 Induces the Ubiquitination of NS3

To determine whether TRIM38 can mediate ubiquitination of NS3, 293T cells were co-transfected with plasmid Myc-NS3 together with the plasmids Flag-TRIM38, Flag-TRIM38-ΔRING, or empty vector, in the presence or absence of His-Ub. After 36 h, cells were lysed with RIPA Lysis Buffer (Beyotime, Shanghai, China) containing 50 mM Tris (pH 7.4), 150 mM NaCl, 1% Triton X-100, 1% sodium deoxycholate, and 0.1% SDS. The supernatants were collected after centrifugation, and the ubiquitination level of NS3 was examined using a Co-IP assay. The results showed that the ubiquitination of NS3 was detected when Myc-NS3 was co-transfected with Flag-TRIM38 and His-Ub. However, TRIM38-ΔRING did not cause significant ubiquitination of NS3 (Figure 6J). These results indicated that TRIM38 promotes the ZIKV NS3 ubiquitination.

## 4. Discussion

The expression of TRIM38 protein is modulated by various viral infections. The expression of TRIM38 protein was significantly increased in primary macrophages infected with Sendai virus (SeV) [14]. Studies found that TRIM38 expression was significantly increased in peripheral blood mononuclear cells (PBMCs) of HBeAg-negative chronic hepatitis B patients, suggesting a potential association with the early treatment effects of peg-IFN-α and HBsAg clearance [15]. Our results showed that ZIKV infection markedly increased both the mRNA and protein levels of TRIM38 in U251 cells, and IFN-β upregulated TRIM38 expression in a dose-dependent manner, suggesting that TRIM38 may function as an ISG involved in the innate immune response against ZIKV.

Studies have found that TRIM38 overexpression significantly reduced HBV mRNA, pgRNA, HBsAg, and HBeAg levels in HepG2.2.15 cells, while TRIM38 knockdown increased these levels. This indicates that TRIM38 can inhibit HBV replication. The antiviral proteins MX1, IFIT1, and STAT1 were upregulated in HepG2.2.15 cells co-treated with IFN-α and TRIM38 compared to those treated with IFN-α alone, indicating that TRIM38 can enhance the antiviral effect of IFN-α [13]. One study found that TRIM25 overexpression in A549 cells could significantly reduce the viral titer of ZIKV. TRIM25 knockdown led to a slight increase in the RNA load of ZIKV but did not significantly affect the viral titer [16]. In this study, TRIM38 overexpression in U251 cells markedly inhibited ZIKV proliferation. In contrast, TRIM38 knockout in U251 cells promoted ZIKV proliferation. Our findings demonstrated that TRIM38 exerts an inhibitory effect on ZIKV proliferation.

The IFN-I response is critical for controlling viral infections and is initiated by the recognition of pathogen-associated molecular patterns (PAMPs) by pathogen recognition receptors (PRRs), such as RIG-I and MDA5. Activated by viral RNA, RIG-I interacts with MAVS to recruit TANK-binding kinase 1 (TBK1) and IκB kinase ε (IKKε), leading to the phosphorylation of interferon regulatory factor 3 (IRF3) and/or IRF7 and their subsequent translocation to the nucleus, which triggers IFN-I expression [17]. TRIM25 can induce oligomerization of RIG-I by modifying its CARDs with K63-linked polyubiquitin and facilitating its binding to MAVS, thereby triggering the expression of downstream antiviral genes to promote IFN-I production [18]. TRIM4 promotes virus-triggered IFN-I responses by mediating K63-linked ubiquitination of RIG-I. TRIM4 overexpression potentiates SeV-triggered activation of IRF3, NF-κB, and the IFN-β promoter, while TRIM4 knockdown attenuates these activations [19]. TRIM38 catalyzes the sumoylation of RIG-I and MDA5, antagonizing their K48-linked polyubiquitination and degradation, thereby enhancing their stability and improving the antiviral innate immune response [11]. In this study, TRIM38 overexpression in U251 cells increased the expression of RIG-I/MDA5 signaling pathway-related proteins, including RIG-I, MDA5, TBK1, and IRF3, at 6 h post-ZIKV infection, and also increased the IFN-β level. At 36 h post-ZIKV infection, the expression levels of interferon-induced antiviral proteins, including ISG15, ISG56, OAS1, OAS2, and OAS3, were increased by TRIM38 overexpression and decreased by TRIM38 knockout. We hypothesize that the upregulation of these ISGs may be attributed to increased IFN-β levels or enhanced efficiency in IFN-β-induced ISG production mediated by TRIM38, and this mechanism warrants further investigation. However, in uninfected cells, except for RIG-I and MDA5, the expression of other proteins did not change significantly, which may be attributed to their extremely low expression levels. During the later stages of infection, TRIM38 negatively regulates the TLR3/4 signaling, consequently suppressing the production of proinflammatory cytokines including TNF-α, IL-1β, and IL-6. This mechanism serves to prevent excessive and detrimental inflammatory responses during the later stages of infection [20]. TRIM38 promotes the polyubiquitination and proteasomal degradation of NF-κB–activating kinase-associated protein 1 (NAP1), thereby negatively regulating TLR- and RIG-I-mediated IFN-β production and antiviral responses [14]. We propose that TRIM38 may exert bidirectional regulation on innate immune signaling in response to distinct viral infections or at different stages of infection.

Some TRIM proteins can exhibit antiviral effects by interacting with viral proteins. TRIM25 degrades the HBx protein of HBV and the VP3 protein of IBDV in a proteasome-dependent manner [21,22], and it degrades the P protein of RABV via the autophagy pathway to inhibit viral replication [23]. At present, few TRIM proteins have been reported to interact with flaviviruses. TRIM52 has been shown to interact with NS2A of JEV and degrade NS2A in a proteasome-dependent manner through its E3 ligase activity [24]. TRIM22 inhibits ZIKV replication by interacting with ZIKV NS1 and NS3 proteins, leading to their degradation through the ubiquitin–proteasome pathway [25]. TRIM79α can mediate lysosome-dependent degradation of the NS5 protein of tick-borne encephalitis virus (TBEV) to restrict viral replication [26]. According to current research, TRIM proteins mediate viral protein degradation primarily through the proteasome pathway and occasionally through the lysosome pathway. In this study, the results of the Co-IP assay showed that TRIM38 interacts with ZIKV NS3 protein, and the immunofluorescence assay demonstrated that both TRIM38 and NS3 co-localize to the cytosol in 293T cells. However, the direct interaction between TRIM38 and NS3, as well as the existence of intermediate molecules between them, needs to be further confirmed by GST pull-down. The TRIM38- and NS3-overexpressing plasmids were co-transfected into 293T cells. With increasing doses of TRIM38, the NS3 protein was degraded. However, this degradation was reversed after treatment with inhibitors NH4Cl and chloroquine, indicating that TRIM38 mediates NS3 protein degradation via a lysosome-dependent pathway. Additionally, in our Co-IP experiment (Figure 5A), the ZIKV C protein interfered with TRIM38 expression, possibly making their interaction difficult to detect. Further investigation is needed to determine whether the C protein interacts with TRIM38 and promotes its degradation.

The NS3 protein is highly conserved among flaviviruses (WNV, JEV, DENV, ZIKV, and YFV), with approximately 67% sequence homology. NS3 plays a crucial role in viral RNA replication, polyprotein cleavage, and virion assembly [27]. ZIKV NS3 is a multifunctional protein containing two functionally distinct domains: an N-terminal domain of 176 amino acids with protease activity and a C-terminal domain of 444 amino acids with helicase, nucleoside 5′-transferase (NTPase), and 5′-terminal RNA triphosphatase (RTPase) activities [28]. The NS2B-NS3 protease complex of ZIKV and DENV mediates the cleavage of the ER-localized reticulophagy receptor FAM134B to inhibit reticulophagy, thereby promoting viral replication [29]. ZIKV NS3 binds to the scaffolding proteins 14-3-3ε and 14-3-3η to block cytosol-to-mitochondria translocation of the sensors RIG-I and MDA5, thereby inhibiting antiviral signaling [30]. We propose that the TRIM38-mediated degradation of NS3 protein may serve as an important mechanism in inhibiting ZIKV replication.

The RING domain of TRIM proteins is composed of 40–60 amino acids and usually confers E3 ubiquitin ligase activity, which can mediate the ubiquitination or sumoylation of specific substrates, thereby playing a critical role in both the degradation of target proteins and innate immune signaling pathways [9]. TRIM52 degrades JEV NS2A protein through the ubiquitin–proteasome pathway, while deletion of the RING domain restores both NS2A protein levels and viral titers. This suggests that the RING domain of TRIM52 is required to mediate ubiquitin-dependent degradation of the NS2A protein and inhibit JEV replication [24]. One study showed that, in contrast to wild-type TRIM38, the TRIM38 mutant with RING deletion exhibited no ubiquitination activity [31]. In this study, we constructed a TRIM38 mutant with RING deletion, Flag-TRIM38-ΔRING (deletion from residues 9 to 66). The Co-IP results demonstrated that TRIM38-ΔRING exhibited negligible ubiquitination activity compared to wild-type TRIM38. Additionally, TRIM38-ΔRING failed to interact with ZIKV NS3 and exhibited no significant antiviral activity, suggesting that the RING domain is essential for the antiviral activity of TRIM38. Growth curves of ZIKV showed that the antiviral efficacy of TRIM38 was stronger during the later stages of infection than during the early stages (Figure 2F and Figure 3F). IFN primarily restricts viruses during the early stages of infection. We propose that the interaction between TRIM38 and ZIKV NS3 protein exerts a more significant direct antiviral effect on ZIKV replication post-infection.

## 5. Conclusions

This study demonstrates that TRIM38 overexpression inhibits ZIKV replication, whereas TRIM38 knockout enhances ZIKV replication in cells. TRIM38 overexpression upregulates the RIG-I/MDA5 pathway and increases IFN-β levels early during viral infection. Furthermore, TRIM38 interacts with the ZIKV NS3 protein and mediates its degradation through a lysosome-dependent pathway. The RING domain is essential for the interaction between TRIM38 and ZIKV NS3, and for inhibiting ZIKV replication. In summary, our study demonstrates that TRIM38 exerts antiviral activity against ZIKV by upregulating the RIG-I/MDA5 pathway, increasing IFN-β levels, and degrading the viral NS3 protein.

## Figures and Tables

**Table 1:** Primers for quantitative RT-PCR.

Gene	Primer	Sequence (5′–3′)
E of ZIKV	P1	GGTTCCACGACATTCCATTAC
	P2	ACTGCTCCTTCTTGACTCCCT
NS3 of ZIKV	P3	GCGTGAGGAACGGCAATGAG
	P4	TGTGACAGGCATGGGTCCAG
IFN-β	P5	GTCTCCTCCAAATTGCTCTCC
	P6	CCACAGGAGCTTCTGACACTG
GAPDH	P7	TCAAGAAGGTGGTGAAGCAGG
	P8	AGCGTCAAAGGTGGAGGAGTG
RIG-I	P9	GACCCTGGACCCTACCTACA
	P10	CCAACTTTCAATGGCTTCAT
MDA5	P11	CCGCTATCTCATCTCGTGCTT
	P12	GTGCCAGACTCCCTTCTCCAA
TBK1	P13	TCTGGTGCAATATCTGGAGTAC
	P14	GAGCTGTCATTTGTTGTAGCG
IRF3	P15	GCCGAGGCCACTGGTGCATAT
	P16	TGGGTCGTGAGGGTCCTTGCT
ISG15	P17	CGCAGATCACCCAGAAGATT
	P18	GCCCTTGTTATTCCTCACCA
ISG56	P19	TTCGGAGAAAGGCATTAGA
	P20	TCCAGGGCTTCATTCATAT
OAS1	P21	GTCAGTTGACTGGCGGCTAT
	P22	CGCTGCTTCAGGAAGTCTCT
OAS2	P23	TGATGTGCTTCCTGCCTTTA
	P24	CCCTTTGGCTTCAGTTTCCT
OAS3	P25	GAAGGAGTTCGTAGAGAAGGCG
	P26	GCATCTCACTGAGGATCTCTGC

## Data Availability

All data are available on reasonable request.
